# Draft Genome Sequence of *Pediococcus parvulus* 2.6, a Probiotic β-Glucan Producer Strain

**DOI:** 10.1128/genomeA.01381-16

**Published:** 2016-12-15

**Authors:** Adrián Pérez-Ramos, M. Luz Mohedano, Ana Puertas, Antonella Lamontanara, Luigi Orru, Giuseppe Spano, Vittorio Capozzi, M. Teresa Dueñas, Paloma López

**Affiliations:** a Centro de Investigaciones Biológicas, C.S.I.C., Madrid, Spain; b Department of Applied Chemistry, University of Basque Country (UPV/EHU), San Sebastián, Spain; c Council for Agricultural Research and Economics (CREA)-Genomics Research Centre, Fiorenzuola d’Arda (PC), Italy; d Department of Agriculture, Food and Environment Sciences, University of Foggia, Foggia, Italy

## Abstract

We report here the draft genome sequence of the probiotic *Pediococcus parvulus* 2.6, a lactic acid bacterial strain isolated from ropy cider. The bacterium produces a prebiotic and immunomodulatory exopolysaccharide, and this is the first strain of the *P. parvulus* species whose genome has been characterized.

## GENOME ANNOUNCEMENT

We report here the draft genome sequence of *Pediococcus parvulus* 2.6 (formerly *Pediococcus damnosus*), a lactic acid bacterium isolated from ropy cider (1). This is the first strain of *P. parvulus* whose genome has been characterized. The 2.6 strain produces the immunomodulatory exopolysaccharide 2-substituted (1,3)-β-d-glucan (2), which is synthesized by a limited number of bacteria and confers probiotic properties to the producing strains. This polysaccharide differs from the β-glucans produced by plants and other microorganisms. The synthesis of 2-substituted (1,3)-β-d-glucan by *P. parvulus* 2.6 is controlled by a single heterotransmembrane glucosyltransferase (GTF), which polymerizes glucosyl residues from UDP glucose (3–5). Some of the conditions that influence the homopolysaccharide (HoPS) synthesis by *P. parvulus* 2.6, as well as the enzymatic activities involved in sugar metabolism in this strain, have been examined (6, 7). The physicochemical properties of the HoPS have also been determined, and nuclear magnetic resonance (NMR) analysis showed that the same HoPS was synthesized, irrespective of the sugar source used for growth (8). The molecular masses of these β-glucans are high (>10^6^ Da), and their rheological properties showed that they have potential utility as biothickeners (8). An oat-based product fermented by *P. parvulus* 2.6 showed improved rheology (9), thereby decreasing the need for added stabilizers and texturizers.

The potential of *P. parvulus* 2.6 as a probiotic strain has also been examined; it resists gastrointestinal stress, adheres to Caco-2 cells, and its HoPS reduces the production of inflammatory cytokines by polarized macrophages (10). We have also shown that *P. parvulus* HoPS improves the growth and viability of probiotic microorganisms, as well as their adhesion to human enterocytes (11). The 2-substituted (1,3)-β-d-glucan increases *in vitro* the ratio of interleukin 10 (IL10) (anti-inflammatory) to tumor necrosis factor alpha (TNFα) (inflammatory) in human macrophages and decreases the levels of the proinflammatory IL8 in human intestine organ cultures (reference 12 and Notararigo S., Antolin M., Guarner F., López P., unpublished data).

An oat-based product fermented by *P. parvulus* 2.6 had a bifidogenic effect and decreased serum cholesterol levels in humans (13). Possibly, the 2-substituted (1,3)-β-d-glucan has a synergistic effect with the hypocholesterolemic action of oat β-glucans. Finally, *P. parvulus* 2.6 displays antibacterial activity against several bacterial species (14), including natural contaminants in oats, a property that reduces the need for chemical preservatives and improves the functionality of the final product.

Two micrograms of genomic DNA was subjected to library preparation using the TruSeq DNA sample perp kit FC-121-1001, according to the manufacturer’s instructions. Whole-genome sequencing used the Illumina GAIIx at the Genomics Research Centre (Fiorenzuolad’Arda, Italy). A total of 26,018,224 paired-end reads (2 × 110-bp length) were assembled into 115 contigs. The genome was calculated to be 2,236,754 long. The size of the shortest contig was 206 bp, while the length of the longest contig was 171,226 bp. The genome sequence was annotated by the NCBI Prokaryotic Genomes Annotation Pipeline. A total of 2,241 genes were predicted to encode 2,069 proteins, three rRNAs, 60 tRNAs, and four noncoding RNAs (ncRNAs), and 105 are pseudogenes.

### Accession number(s).

The complete genome of *P. parvulus* 2.6 has been deposited at DDBJ/EMBL/GenBank under accession number LXND00000000.

## References

[1] Fernández K, Dueñas M, Irastorza A, Bilbao A, del Campo G 1995 Characterization and DNA plasmid analysis of ropy *Pediococcus* spp. strains isolated from Basque Country ciders. J Food Protect 59:35–40. 10.4315/0362-028X-59.1.35 31158968

[2] Dueñas-Chasco MT, Rodríguez-Carvajal MA, Mateo PT, Franco-Rodríguez G, Espartero J, Irastorza-Iribas A, Gil-Serrano AM 1997 Structural analysis of the exopolysaccharide produced by *Pediococcus damnosus* 2.6. Carbohydr Res 303:453–458. doi:10.1016/S0008-6215(97)00192-4. 9403990

[3] Werning ML, Ibarburu I, Dueñas MT, Irastorza A, Navas J, López P 2006 *Pediococcus parvulus gtf* gene encoding the GTF glycosyltransferase and its application for specific PCR detection of β-d-glucan-producing bacteria in foods and beverages. J Food Prot 69:161–169. 16416914 10.4315/0362-028x-69.1.161

[4] Werning ML, Corrales MA, Prieto A, de Palencia PF, Navas J, López P 2008 Heterologous expression of a position 2-substituted (1→3)-β-d-glucan in *Lactococcus lactis*. Appl Environ Microbiol 74:5259–5262. doi:10.1128/AEM.00463-08. 18567684 PMC2519285

[5] Werning ML, Pérez-Ramos A, Fernández de Palencia P, Mohedano ML, Dueñas MT, Prieto A, López P 2014 A specific immunological method to detect and quantify bacterial 2-substituted (1,3)-β-d-glucan. Carbohydr Polym 113:39–45. doi:10.1016/j.carbpol.2014.06.072. 25256456

[6] Velasco S, Arsköld E, Paese M, Grage H, Irastorza A, Rådström P, van Niel EW 2006 Environmental factors influencing growth of and exopolysaccharide formation by *Pediococcus parvulus* 2.6. Int J Food Microbiol 111:252–258. doi:10.1016/j.ijfoodmicro.2006.06.008. 16854485

[7] Velasco SE, Yebra MJ, Monedero V, Ibarburu I, Dueñas MT, Irastorza A 2007 Influence of the carbohydrate source on β-glucan production and enzyme activities involved in sugar metabolism in *Pediococcus parvulus* 2.6. Int J Food Microbiol 115:325–334. doi:10.1016/j.ijfoodmicro.2006.12.023. 17303279

[8] Velasco SE, Areizaga J, Irastorza A, Dueñas MT, Santamaria A, Muñoz ME 2009 Chemical and rheological properties of the β-glucan produced by *Pediococcus parvulus* 2.6. J Agric Food Chem 57:1827–1834. doi:10.1021/jf803065w. 19219990

[9] Mårtensson O, Staaf J, Dueñas-Chasco MT, Irastorza A, Oste R, Holst O 2002 A fermented ropy non-dairy oat product based on the exopolysaccharideproducing strain *Pediococcus damnosus* 2.6. Adv Food Sci 24:4–11. doi:10.1016/j.nutres.2005.03.004.

[10] de Palencia PF, Werning ML, Sierra-Filardi E, Dueñas MT, Irastorza A, Corbí AL, López P 2009 Probiotic properties of the 2-substituted (1,3)-β-d-glucan producing *Pedioccus parvulus* 2.6. Appl Environ Microbiol 75:4887–4891. doi:10.1128/AEM.00394-09. 19465528 PMC2708447

[11] Russo P, López P, Capozzi V, de Palencia PF, Dueñas MT, Spano G, Fiocco D 2012 Beta-glucans improve growth, viability and colonization of probiotic microorganisms. Int J Mol Sci 13:6026–6039. doi:10.1128/AEM.00394-09. 22754347 PMC3382753

[12] Notararigo S, de las Casas-Engel M, Fernández de Palencia P, Corbí AL, López P 2014 Immunomodulation of human macrophages and myeloid cells by 2-substituted (1,3)-β-d-glucan from *P. parvulus* 2.6. Carbohydr Polym 112:109–113. doi:10.1016/j.carbpol.2014.05.073. 25129723

[13] Mårtensson O, Biörklund M, Lambo AM, Dueñas-Chasco M, Irastorza A, Holst O, Norin E, Welling G, Öste R, Önning G 2005 Fermented ropy, oat-based products reduce cholesterol levels and stimulate the bifidobacteria flora in humans. Nutr Res 25:429–442. doi:10.1016/j.nutres.2005.03.004.

[14] Immerstrand T, Paul CJ, Rosenquist A, Deraz S, Mårtensson OB, Ljungh A, Blücher A, Öste R, Holst O, Karlsson EN 2010 Characterization of the properties of *Pediococcus parvulus* for probiotic or protective culture use. J Food Prot 73:960–967. 20501049 10.4315/0362-028x-73.5.960

